# Low level of interobserver concordance in assessing histological subtype and tumor grade in patients with penile cancer may impair patient care

**DOI:** 10.1007/s00428-021-03249-5

**Published:** 2021-12-10

**Authors:** Luiza Dorofte, Diane Grélaud, Michelangelo Fiorentino, Francesca Giunchi, Costantino Ricci, Tania Franceschini, Mattia Riefolo, Sabina Davidsson, Jessica Carlsson, Gabriella Lillsunde Larsson, Mats G. Karlsson

**Affiliations:** 1 Department of Laboratory Medicine, Faculty of Medicine and Health, Örebro University, Örebro, Sweden; 2 Department of Clinical Genetics and Pathology, Skåne University Hospital and Regional Laboratories, Malmö, Sweden; 3 Department of Experimental, Diagnostic and Specialty Medicine, Alma Mater Studiorum, University of Bologna, Bologna, Italy; 4 Pathology, Istituto Di Ricovero E Cure a Carattere Scientifico, Azienda Ospedaliero-Universitaria Policlinico Sant’Orsola-Malpighi, Bologna, Italy; 5 Pathology Unit, Maggiore Hospital, AUSL Bologna, Bologna, Italy; 6 Department of Urology, Faculty of Medicine and Health, Örebro University, Örebro, Sweden; 7 School of Health Sciences, Örebro University, Örebro, Sweden

**Keywords:** Penile cancer, Interobserver agreement, Histological grading, Penile carcinoma subtypes

## Abstract

Differentiation between penile squamous cell carcinoma patients who can benefit from limited organ-sparing surgery and those at significant risk of lymph node metastasis is based on histopathological prognostic factors including histological grade and tumor histological subtype. We examined levels of interobserver and intraobserver agreement in assessment of histological subtype and grade in 207 patients with penile squamous cell carcinoma. The cases were assessed by seven pathologists from three hospitals located in Sweden and Italy. There was poor to moderate concordance in assessing both histological subtype and grade, with Fleiss kappas of 0.25 (range: 0.02–0.48) and 0.23 (range: 0.07–0.55), respectively. When choosing HPV-associated and non-HPV-associated subtypes, interobserver concordance ranged from poor to good, with a Fleiss kappa value of 0.36 (range: 0.02–0.79). A re-review of the slides by two of the pathologists showed very good intraobserver concordance in assessing histological grade and subtype, with Cohen’s kappa values of 0.94 and 0.91 for grade and 0.95 and 0.84 for subtype. Low interobserver concordance could lead to undertreatment and overtreatment of many patients with penile cancer, and brings into question the utility of tumor histological subtype and tumor grade in determining patient treatment in pT1 tumors.

**Supplementary Information:**

The online version contains supplementary material available at 10.1007/s00428-021-03249-5.

## Introduction

Penile cancer is a rare malignancy, especially in developed countries. The annual age-standardized global incidence is 0.84 cases per 100,000. However, variations in incidence exist between different countries, likely depending on differences in lifestyle and local practices regarding hygiene, social behavior, and religion [1, 2]. Known risk factors for penile cancer include human papillomavirus (HPV) infection, phimosis, lichen sclerosus and other inflammatory conditions, UVA phototherapy, smoking, and socioeconomic status [3–5]. Circumcision has been associated with a reduced risk of penile cancer [4, 6, 7].

A few different histological subtypes of penile squamous cell carcinoma have been described earlier in the literature, but the first report of a clear correlation between certain histological subtypes of invasive penile carcinoma and HPV infection as well as the first histological subclassification was published in 1995 by Gregoire et al. [8]. Approximately 50% of cases of penile cancer are associated with HPV infection [9, 10]. The role of HPV in the carcinogenesis of anogenital tumors and tumors of the head and neck area has been known for a long time. It has been demonstrated that patients with HPV-associated squamous cell carcinoma of the head and neck region have a better prognosis than those with HPV-negative tumors [11–13]. Recent studies reported that patients with a HPV-positive penile tumor had better recurrence-free survival rates [9, 14–16]. Moreover, tumors that showed an HPV-associated morphology also had lower risk of lymph node metastasis [9]. Multiple histological subtypes of penile squamous cell carcinoma have been added to the classification over the years, and are included in the 2016 World Health Organization (WHO) classification of tumors [17] under two major categories: HPV-related and non-HPV-related subtypes. HPV-associated tumors are usually high grade, most often with basaloid or warty histological subtype. HPV-negative tumors are most often associated with inflammatory conditions such as lichen sclerosus et atrophicus and lichen planus, and usually show a verrucous or usual histological subtype.

The main prognostic factor involved in patient survival is represented by the presence of lymph node metastasis [18]. Approximately 12–25% of patients without clinically palpable lymph nodes will develop occult metastases [19, 20]. Due to the fact that inguinal lymph node dissection (ILND) can be associated with serious complications and increased mortality, predictive histological factors have been used in choosing the best treatment option. It has been shown that tumor histological grade and stage are the most important prognostic elements for prediction of lymph node metastasis in penile cancer patients with clinically negative lymph nodes [19, 21]. In the TNM classification of malignant tumors (TNM), the staging system for penile cancer has been revised multiple times since it was introduced in 1968 [20, 22–24]. The histological tumor grade has been included in the latest TNM classification for penile cancer as a criterion for inclusion in the pT1a and pT1b subclasses [24]. According to the WHO and the International Society of Urological Pathology (ISUP), most of the different histological subtypes of penile cancer are associated with certain histological grades and show a different risk for nodal metastasis [17, 25]. The European Association of Urology recommends ILND in primary penile tumors pT1G2, G3 as well as tumors pT2 and pT3. Patients with low-risk tumors (pTa, pTis, or pT1aG1) benefit from organ-sparing surgery and no ILND is needed [26].

Correct identification of histological subtypes that present a higher risk for lymph node metastasis and correct assessment of tumor histological grade are of great importance in management of patients with penile cancer, in order to avoid unnecessary ILND. As penile cancer is a rare type of tumor, most pathologists are not well acquainted with different subtypes and tumor grading. To our knowledge, only a few studies on interobserver reproducibility in assessing the histological tumor grade have been published [27–29], and there has been no report on interobserver variation in assessing the histological subtypes of penile cancer.

The aim of this study was to evaluate the levels of interobserver and intraobserver concordance in assessing histological subtype and grade of penile squamous cell cancer, as well as the impact of subjectivity in assessing grade and tumor subtype when choosing treatment management.

## Materials and methods

### Study cases

We retrospectively reviewed tissue specimens from 229 consecutive patients who underwent surgical treatment for penile cancer between 2009 and 2016 at Örebro University Hospital, Sweden. From these 229 cases, we excluded cases that had limited tumor material (*n* = 3) as well as cases that included only large glass slides that could not be scanned in our current slide scanner (*n* = 19). Overall, tissues from a total of 207 cases were included in our study. The study was approved by the Swedish Ethical Review Authority (2019–01923).

### Study design

After patient identification in the laboratory informatics system and retrieval of material from the local hospital archive, all the cases were assessed on hematoxylin–eosin stain by a local pathologist. Representative slides from each tumor were chosen and scanned using the Panoramic 250 Flash II system (3DHISTECK, Budapest, Hungary). All the scanned slides were converted into high-resolution digital slides, and the histological evaluation was performed using version 2.1 of the Case Viewer software package (3DHISTECK). The digital slides were evaluated by three experienced pathologists subspecializing in uropathology, a recent specialist, and three residents in pathology from three hospitals in Sweden and Italy. Two of the pathologists (graders 1 and 7) are subspecialized in the diagnosis of penile cancer, and work in hospitals specializing in the treatment of penile cancer. The following variables were assessed: tumor histological subtype and tumor histological grade. All pathologists followed the histological criteria recommended by WHO classification of tumors [17] and its guidelines for grading of penile cancer. Information regarding the presence/absence of HPV in the tumor material was unknown to the pathologists, in order to avoid subjective assessment of the histological tumor subtype. Two of the most experienced pathologists (graders 1 and 2) also re-examined the digital slides a month after the first evaluation in order to assess the level of intraobserver agreement.

### Tumor histological subtype

**Fig. 1:**
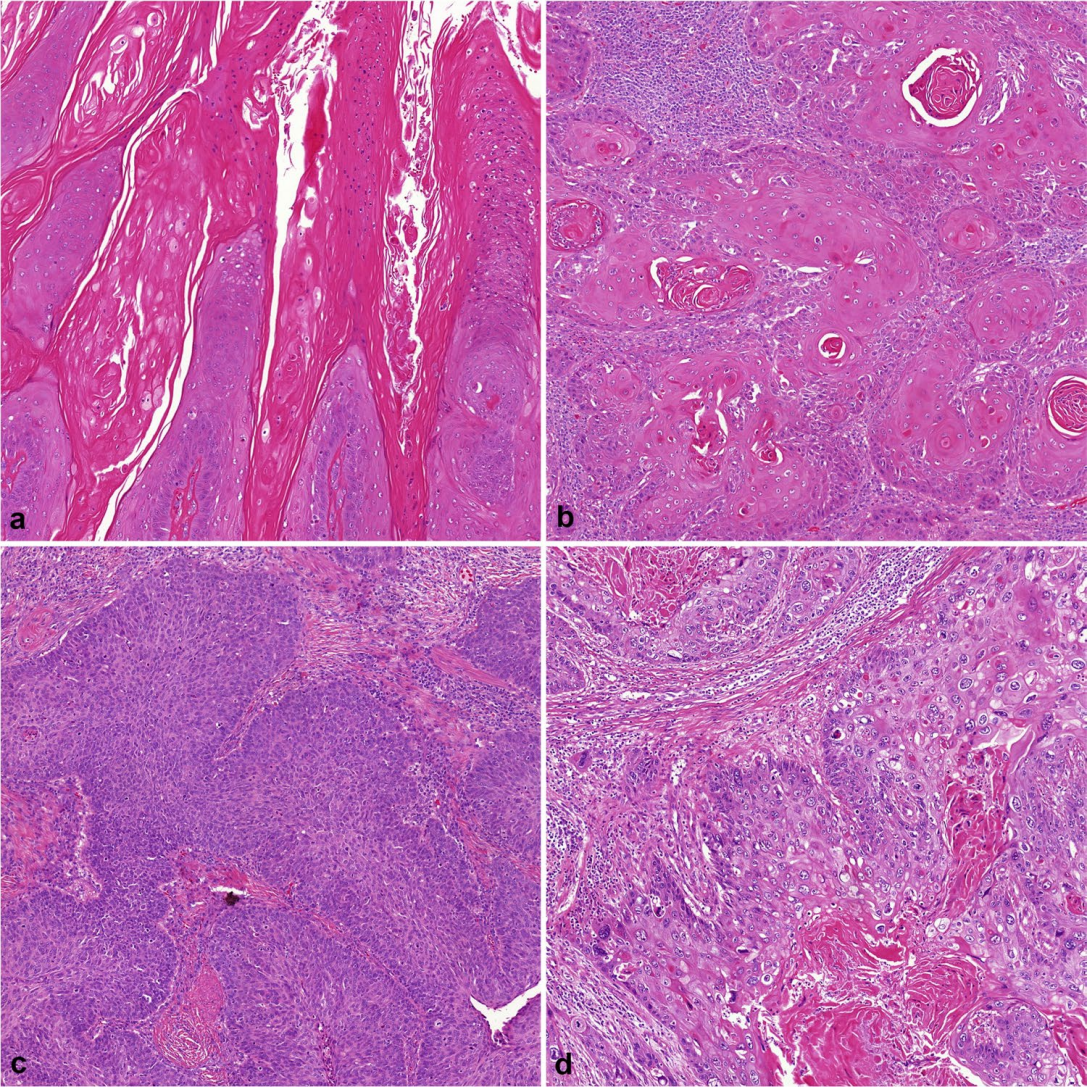
Subtypes of penile squamous cell carcinoma: hematoxylin–eosin stain, magnification × 100. **a** Verrucous subtype, **b** usual subtype, **c** basaloid subtype, **d** warty subtype

The histological subtype of primary penile tumors (Fig. 1) was assessed according to criteria published by the WHO classification of tumors of the urinary system and ISUP recommendation (2016) [17, 25]. According to this classification, penile squamous cell carcinoma is subclassified in twelve different histological subtypes divided into two categories. Non-HPV-related carcinomas include the following subtypes: usual, verrucous with its variants, carcinoma cuniculatum and pseudohyperplastic, pseudoglandular, papillary, adenosquamous, sarcomatoid as well as mixed forms, most often hybrid verrucous carcinomas. The HPV-related carcinoma category includes basaloid, warty, clear cell, lymphoepithelioma-like carcinoma, and mixed (most often warty-basaloid) subtypes.

### Histological grade

**Fig. 2:**
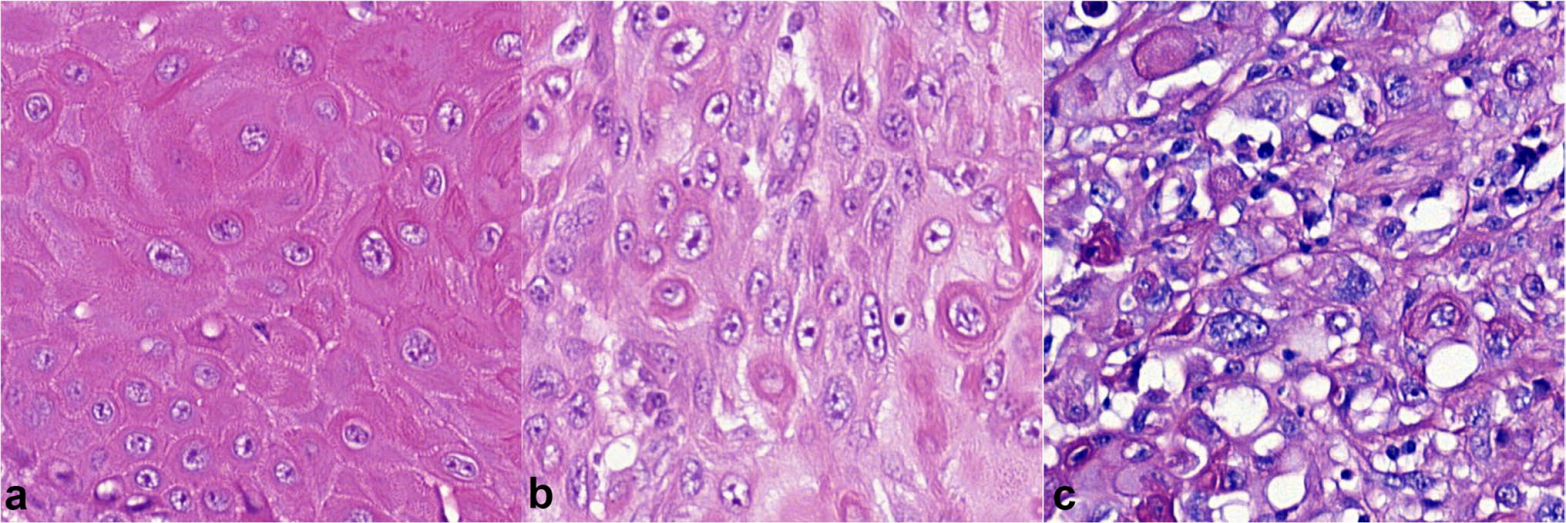
Histopathologic grading of penile squamous cell carcinoma according to WHO recommendations: hematoxylin–eosin stain, magnification × 400. **a** Grade 1, **b** grade 2, **c** grade 3

The tumors were graded using a three-tiered system based on ISUP/WHO recommendations [17, 25]. In this system, grade 1 tumors are well differentiated with minimal cell atypia, and grade 3 tumors show no maturation and have a high cell pleomorphism and high mitotic activity. Tumors that do not meet the criteria for grades 1 or 3 belong to grade 2 (Fig. 2). Squamous cell carcinomas are usually heterogeneous and show different grades in different parts of the tumor; the histological grade is assigned based on the highest observed grade, as any proportion of high grade is relevant.

### Statistical analysis

Interobserver and intraobserver concordance were assessed by calculating Cohen’s kappa (κ) between pairs of observers for histological grade, subtype, and inclusion in the HPV-positive or HPV-negative group. Cohen’s κ was calculated using version 25 of IBM SPSS Statistics (IBM Corp., Armonk, NY, USA), and Fleiss’ κ for overall concordance among all seven graders was calculated in R [30], using the package irr [31]. The degree of concordance was classified as poor at κ values of 0.00–0.20, fair at κ values of 0.21–0.40, moderate at κ values of 0.41–0.60, good at κ values of 0.61–0.80, and very good at κ values of 0.81–1.00 [32].

## Results

### Histological subtype

The level of concordance in assessing tumor histological subtype was fair (Table 1, upper right), with a Fleiss’ κ of 0.25 (Cohen’s κ values between 0.02 and 0.48). All twelve histological subtypes were identified, as well as eleven mixed forms (*n* = 23).

**Table 1:**
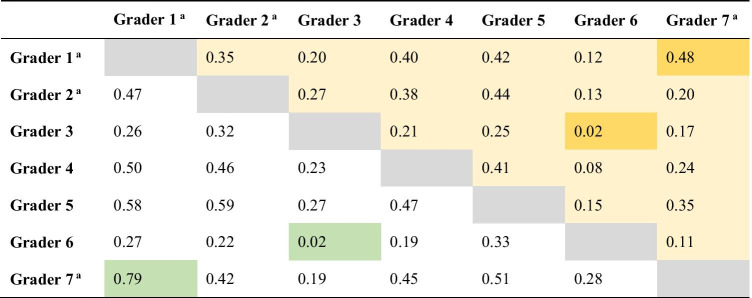
Cohen’s kappa values for interobserver concordance on histological subtyping (upper right) and human papillomavirus (HPV)-associated and non-HPV-associated subtypes (lower left)

^a^Pathologists with most experience in uropathology

When analyzing HPV-associated and non-HPV-associated subtypes (*n* = 2) (Table 1, lower left), the interobserver concordance was fair, with a Fleiss’ κ value of 0.36 (Cohen’s κ values between 0.02 and 0.79). Tumors with mixed morphology that included both HPV-associated and non-HPV-associated subtypes were included in the HPV-associated tumor group. The histological subtypes with best interobserver concordance using Fleiss’ κ were basaloid, sarcomatoid, lymphoepithelioma-like, and usual (κ values of 0.51, 0.50, 0.83, and 0.33, respectively). For mixed histological subtypes, there was poor to fair concordance, with Fleiss’ κ values ranging from − 0.001 to 0.036. The best concordance in assessing tumor histological subtype in general was seen between the pathologists specializing in diagnosis of penile tumors (graders 1 and 7).

Graders 1 and 2 also re-reviewed the scanned slides in order to assess the intraobserver agreement on histological subtype. There was a very good concordance between the first review and the re-review, with Cohen’s κ values of 0.95 and 0.81, respectively, for the two graders (intraobserver agreement of 96.6% and 88.4%).

### Histological grade

The interobserver agreement in histological tumor grade (Table 2) showed a fair concordance, with a Fleiss’ κ of 0.23 (Cohen’s κ values between 0.07 and 0.55). The highest level of concordance for grade was found between the most experienced pathologists.

**Table 2:**
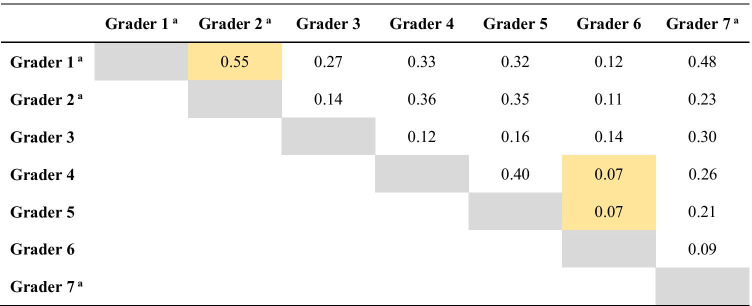
Cohen’s kappa values for interobserver concordance on histologic grade

^a^Pathologists with most experience in uropathology

The intraobserver agreement between graders 1 and 2 in assessing tumor grade was 94.2% and 96.1%, respectively. The concordance was very good, with Cohen’s κ values of 0.94 and 0.91.

When assessing grade, the different pathologists graded 2.4–34.3% of the evaluated tumors as G1 tumors, 21.3–70.0% as G2 tumors, and 27.5–62.8% as G3 tumors. Three of the seven pathologists graded more than 50% of the tumors as grade 2, while two graders (4 and 5) graded more than 50% of the tumors as grade 3. The last grader (grader 2) had an equal distribution of grade 1–3 tumors (Table 3).

**Table 3:** Observer results for histological tumor grading

Grade	Grader 1^a^	Grader 2^a^	Grader 3	Grader 4	Grader 5	Grader 6	Grader 7^a^
	*n*	*n*	*n*	*n*	*n*	*n*	*n*
1	45	71	5	35	33	20	26
2	101	61	145	54	44	126	116
3	61	75	57	118	130	61	65
Total	207	207	207	207	207	207	207

^a^Pathologists with most experience in uropathology

## Discussion

When it comes to survival of patients with penile cancer, the presence of inguinal lymph node metastasis is one of the most important prognostic factors. Up to 25% of patients with penile cancer will benefit from ILND. At the same time, the procedure is associated with short- or long-term complications like wound dehiscence, necrosis, infection, lymphedema, and lymphocele in up to 84% of cases [33]. Thus, the identification of patients whose tumors are low or high risk for lymph node metastasis is crucial, in order to avoid both undertreatment and overtreatment.

Previous studies have shown an association between tumor histological subtype and the risk of lymph node metastasis in penile cancer [9, 34–37]. While the verrucous variant of squamous cell carcinoma and its variants pseudohyperplastic and carcinoma cuniculatum have a very low potential for metastasis and an excellent prognosis, the sarcomatous and basaloid subtypes have been associated with an increased risk of lymph node metastasis and mortality [17, 34, 36]. The European Association of Urology guidelines classify the different subtypes of penile cancer into three risk groups based on the risk of lymph node metastasis. The low-risk group consists of the verrucous subtype and its variants, along with the warty and papillary subtypes. The usual and mixed subtypes are included in the intermediate-risk group, and the high-risk group includes basaloid, adeno-squamous, sarcomatoid, and poorly differentiated variant of warty subtypes [26]. In the light of these data, correct identification of the histological subtype plays an important role in assessing the risk of nodal metastasis and in choosing the best treatment. While our data show the best interobserver concordance regarding usual, basaloid, lymphoepithelioma-like, and sarcomatoid subtypes of squamous cell cancer, poor concordance was observed in the mixed subtypes that represent up to 25% of penile tumors [17] and which are included in the intermediate-risk group.

Most of the pathologists participating in this study had a tendency to choose pure subtypes instead of mixed variants. This can have an important impact on the choice of treatment. While verrucous cancer, a low-risk tumor, can in certain cases have a more limited treatment approach with organ-sparing surgery and surveillance of lymph node status, the verrucous-usual mixed subtype is an intermediate-risk tumor which needs a more aggressive local surgical approach and can benefit from dynamic sentinel node biopsy or modified ILND. Our finding of higher levels of interobserver concordance between more experienced pathologists shows the importance of good knowledge of uropathology and good experience as a pathologist in diagnosis of penile tumors. The ability to recognize different histological subtypes included in the different risk groups, which have different treatment approaches, is of great importance [26]. The pathologist should be able to identify the correct subtype and should include it in the pathology report.

Higher survival rates in patients with HPV-related penile cancer, along with the future possibility of different treatment options based on the histological subtype, make a correct assessment of the histological HPV-associated and non-HPV-associated subtypes even more important. Because HPV analysis is not available in many countries with high incidence of penile cancer, a good knowledge of morphological diagnostic criteria is needed. Most of the histological subtypes can be recognized on the basis of characteristic morphological features with no need for HPV analysis, but in difficult cases the WHO recommends the use of p16 immunostaining [25]. Our study shows that pathologists who have experience in working with penile cancer have a good concordance in identifying HPV-related and non-HPV-related histological subtypes of squamous cell carcinoma. Further studies of the histological tumor characteristics and the role of HPV infection in carcinogenesis might lead to better and more objective prognostic factors for patients with penile cancer.

The latest TNM classification from 2016 includes tumor histological grade in staging of penile cancer. Thus, stage pT1 has been divided into pT1a (histological grade G1–G2 without vascular invasion) and pT1b (histological G3 and/or vascular invasion). According to Solsona et al. [19], there are three risk group categories of penile cancer. The low-risk group comprises tumors at stage pT1/G1; the intermediate-risk group includes tumors pT1, G2–3 and pT2, G1; and the high-risk group consists of tumors pT2, G2–G3 and pT3, pT4 [19]. Patients in the high-risk and intermediate-risk groups with clinically negative inguinal lymph nodes can benefit from ILND as part of standard treatment. Tumor stage and histological grade also play an important role in treatment management of the primary tumor, with possibilities of different techniques of organ-sparing surgery for tumors pT1a and pT1b [38]. This is especially important in younger patients who are still sexually active, and in whom an organ-sparing surgical treatment with good cosmetic and functional results has an important impact on quality of life.

Our study showed poor to moderate concordance between different pathologists in assessing the histological grade, with a higher level of concordance between experienced pathologists. The broad intervals in percentages of G1, G2, and G3 tumors in individual assessments raises the question of whether risk group categories and the division of pT1 stage should be based on this subjective prognostic factor. For example, in terms of grade distribution (Table 3), G1 tumors represented between 2.4 and 34.3% of the total cases depending on the assessing pathologist. If all the cases were stage pT1, between 5 and 71 patients (total *n* = 207) would benefit from organ-sparing surgery and no ILND would be needed. The question remains of how many of these patients will be undertreated or overtreated given such large variation in the results of histological grading. Histological grading of squamous cell carcinoma lacks a standard grading system, and is highly subjective [27–29, 39]. Studies of interobserver agreement on histological grading of squamous cancer in general have been performed using different grading systems, but all have shown low levels of agreement [27, 28, 39–41]. Among all the different TNM classifications of squamous cell carcinoma in specific locations, only penile cancer has histological grade as part of TNM classification.

The different levels of experience of the reviewers in this study can be seen as a limitation. On the other hand, our study has been able to reveal the importance of both subspecialization in uropathology and experience in assessing grade and tumor subtype when it comes to penile pathology. The major strengths are the large number of cases included and the substantial number of assessing pathologists.

## Conclusions

Our study demonstrates a broad spectrum of interobserver concordance between different pathologists in assessing the histological tumor subtype, in choosing HPV-associated and HPV-negative subtypes of squamous cell carcinoma, and in histological grading of penile cancer. The level of disagreement depends on personal subjectivity, the lack of standardized objective morphologic grading criteria, and level of experience in the diagnosis of penile cancer. The use of tumor histological subtype as a criterion for separation into different risk groups and the division of pT1 stage based on histological grade might not have the best prognostic value when it comes to choosing the right treatment option. Most pathologists rarely have the chance to see cases of penile cancer in daily practice and are not aware of the new classifications and recommendations, and thus lack experience in grading and subtyping such tumors; this, in turn, has an impact on patient management. The higher level of concordance found between the experienced pathologists in this study shows the importance of good knowledge of penile pathology, and of pathology in general, in order to avoid overtreatment or undertreatment of patients with penile cancer. The subspecialty of pathologists surely improves the concordance and the overall quality of the diagnoses in penile pathology. Further studies of tumor biology and etiological factors are needed in order to discover more reliable prognostic factors and for improvement of the diagnosis and treatment of patients with penile cancer.

## Supplementary Information

Below is the link to the electronic supplementary material.

Supplementary file1 (XLSX 32 KB)

## Abbreviations

HPV: Human papillomavirus
ISUP: International Society of Urological Pathology
WHO: World Health Organization
G1–3: Histological grade
TNM: TNM classification of malignant tumors
ILND: Inguinal lymph node dissection
